# Oxidative Stress and Acute Kidney Injury in Critical Illness: Pathophysiologic Mechanisms—Biomarkers—Interventions, and Future Perspectives

**DOI:** 10.1155/2017/6193694

**Published:** 2017-09-28

**Authors:** Paraskevi Pavlakou, Vassilios Liakopoulos, Theodoros Eleftheriadis, Michael Mitsis, Evangelia Dounousi

**Affiliations:** ^1^Department of Nephrology, Medical School University of Ioannina, Ioannina, Greece; ^2^Division of nephrology and Hypertension, 1st Department of Internal Medicine, AHEPA Hospital, School of Medicine, Aristotle University of Thessaloniki, Thessaloniki, Greece; ^3^Department of Nephrology, Medical School, University of Thessaly, Larissa, Greece; ^4^Department of Surgery, Medical School University of Ioannina, Ioannina, Greece

## Abstract

Acute kidney injury (AKI) is a multifactorial entity that occurs in a variety of clinical settings. Although AKI is not a usual reason for intensive care unit (ICU) admission, it often complicates critically ill patients' clinical course requiring renal replacement therapy progressing sometimes to end-stage renal disease and increasing mortality. The causes of AKI in the group of ICU patients are further complicated from damaged metabolic state, systemic inflammation, sepsis, and hemodynamic dysregulations, leading to an imbalance that generates oxidative stress response. Abundant experimental and to a less extent clinical data support the important role of oxidative stress-related mechanisms in the injury phase of AKI. The purpose of this article is to present the main pathophysiologic mechanisms of AKI in ICU patients focusing on the different aspects of oxidative stress generation, the available evidence of interventional measures for AKI prevention, biomarkers used in a clinical setting, and future perspectives in oxidative stress regulation.

## 1. Introduction

Acute kidney injury (AKI) is a multifactorial clinical entity that presents with primary and secondary nonspecific manifestations due to a variety of causes (Table 1). Until the beginning of the 21st century, the incidence of AKI was not accurately reported due to the fact that AKI definition was highly dependent on clinician's opinion and widely varied among different centers [1]. The definition and diagnosis of AKI based on standard criteria were first developed in 2004 by the Second International Consensus Conference of the Acute Dialysis Quality Initiative (ADQI) Group which introduced the RIFLE (Risk, Injury, Failure, Loss, End-stage kidney disease) criteria [2] (Table 1). The different stages in RIFLE classification are delineated according to changes in serum creatinine levels and/or glomerular filtration rate (GFR) or urine output [2]. In 2007, the Acute Kidney Injury Network (AKIN) published a report that established AKI is the term to be used in order to describe the whole spectrum of acute kidney failure and proposed a modified RIFLE classification without including separately renal replacement therapy (RRT) [3]. Most recently, in 2012, Kidney Disease: Improving Global Outcomes (KDIGO) working group proposed that for accuracy purposes, serum creatinine measurements should be used instead of GFR estimation when staging AKI [4] and a guideline report was endorsed by the National Kidney Foundation Kidney Disease Outcomes Quality Initiative (NKF-KDOQI) as well [5] (Table 2).

AKI represents a major public health problem with a reported incidence of 0.25% in the general population and 18% in the hospitalized patients [5]. Although AKI is not a usual cause for admission to intensive care unit (ICU), it often complicates critically ill patients' clinical course. Epidemiologic evidence of all-cause AKI incidence in ICU patients widely varies due to the remarkable polyphony previously used in diagnostic criteria and ranges from 5.7% [6] to 36% [7–9]. The severity of AKI defined by RIFLE classification has been reported to be 36.1% and seems to be an independent risk factor for patients' outcome and mortality [7, 8]. Moreover, in a critical care setting, AKI is connected with prolongation of hospitalization and need for RRT and occasional progress to chronic kidney disease. Sepsis is the leading cause of AKI in severely ill patients in ICU, accounting for nearly 50% of cases [10], while common concurrent diseases further complicate the outcome of these patients including congestive heart failure, liver disease, malignancies, and chronic obstructive pulmonary disease [11] as well as preventable causes that are derived from surgical procedures and prolonged hospitalization [12].

Kidney is a highly vulnerable organ, and the etiology of AKI is of multiple origins. Nevertheless, in the majority of situations, renal parenchyma integrity is disrupted either in terms of hypoperfusion that ends up in renal tubular dysfunction or by direct damage from “toxins” that further injure kidney's interstitial tissue and cellular functions [13]. Oxidative stress gives rise to a chain-like response through direct production of reactive oxygen species (ROS) and metabolic products that act as ligands for receptor types (i.e., toll-like receptors) whose activation is the “alarm” for an ongoing harmful process in AKI. Those circulating “toxins” are inflammatory products that mediate the expansion of injury and hemodynamic imbalance [14]. Critical illness is interwoven with acute inflammation and the consequent production of ROS that feed oxidative stress response. Albeit etiology (hemodynamic dysregulations, infections, rhabdomyolysis, cardiorenal syndrome, uremia, inadequate clearance of metabolism products, etc.), AKI and oxidative stress preserve a bidirectional relationship in critically ill patients. The purpose of this article is to present the main pathophysiologic mechanisms of AKI in ICU patients focusing on the different aspects of oxidative stress generation, the available evidence of interventional measures for AKI prevention, biomarkers used in a clinical setting, and future perspectives in oxidative stress regulation.

### 1.1. Oxidative Stress and Its Pathogenetic Role in AKI

Oxidative metabolism constitutes a fundamental process for aerobic organisms in order to cover energy needs and respond to emergency metabolic situations [15, 16]. Under normal circumstances, the balance between oxidants and antioxidant production is retained in favor of homeostasis. Oxidative stress was introduced for the first time by Stahland and Sies in 1985 [17] and is briefly defined as the metabolic disturbances, such as increased production of oxidants that leads to the depletion of endogenous antioxidants with inadequate decompensation and ends up in cellular damage [15], dysfunction of proteins, and damage of DNA, lipids, and enzymes [17]. The quantification of oxidative stress can be approached only indirectly, by measuring by-products such as isoprostanes [18], malondialdehyde levels [19], and other protein damage markers [20] with techniques and results that have been questioned. On the other hand, endogenous antioxidant systems are self-defense mechanisms with a crucial participation in the maintenance of immune system integrity that are activated when oxidative stress cannot be counterbalanced [21]. When organisms sense a possible threat, they have the ability to delay metabolic processes and even enter cell cycle arrest in order to avoid further oxidative damage.

**Figure 1:**
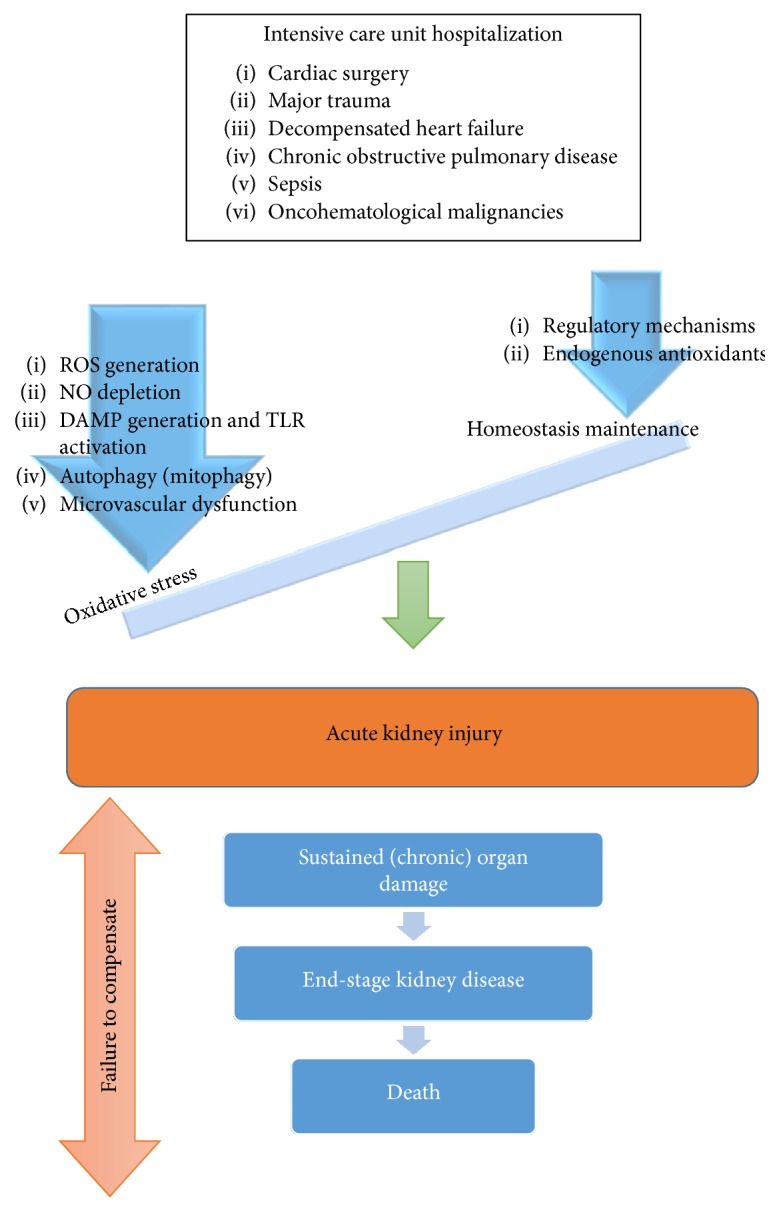
Progress of acute kidney injury in critical illness-associated oxidative stress. Critically ill patients in intensive care units suffer from multifactorial disorders that are added up against the potentiality of regulatory mechanisms to maintain homeostasis, leading to further imbalance in favor of oxidative stress generation through multiple pathogenetic pathways. Once this cataract leads to renal damage with the form of acute kidney injury, the prolonged exposure to oxidative stress environment leads to an uneventful outcome that ranges from chronic kidney disease to death. ROS: reactive oxygen species; NO: nitric oxide; DAMPs: danger-associated molecular patterns.

The pathophysiology of AKI constitutes a complex interplay among vascular, tubular, and inflammatory factors which is followed by a repair process that can either restore epithelial cells and physiological function or result in progressive fibrosis and chronic kidney damage. Abundant experimental and to a less extent clinical data support the important role of oxidative stress-related mechanics in the injury phase of AKI (Figure 1()). The more extensively explored and better-established mechanisms of oxidative stress involved in the pathogenesis of AKI will be reviewed.

#### 1.1.1. Reactive Oxygen Species (ROS) and Nitric Oxide (NO)

Mitochondrion is the primary energy factory of the human body and is abundant in proximal renal tubule making renal cortex a crucial field of oxygen use for energy production. Moreover, in AKI, mitochondrial injury precedes other renal manifestations even the increase of serum creatinine levels [22]. The main source of ROS generation is the reduction of oxygen by cytochrome oxidase in mitochondrial electron chain transport (ETC) that results in the production of hydrogen peroxide (H_2_O_2_), superoxide anion radical (O_2_^−^), and hydroxyl radical (HO) [15]. There is no specific target for ROS, but the attack on lipids, proteins, and amino acids results in the formation of unstable molecules that act as radicals and finally convert into compounds with multiple metabolic effects [16]. Consequently, lipid, protein, and nucleic acid peroxides belong in ROS family. Minor ROS generators (about 10% in total) are xanthine oxidase, NADPH oxidase complex (Nox), and adrenaline/epinephrine as well [15, 23].

Kidney receives about 25% of total blood supply and is rich in mitochondria that render it susceptible to damage from ROS and subsequent development of AKI [24]. Cellular apoptosis, lipid peroxidation, and imbalanced calcium concentration are few of the induced mechanisms by ROS [25]. Two characteristic representatives of AKI are ischemia reperfusion injury (IRI) and sepsis [24]. Aggressive fluid resuscitation for retaining hemodynamic balance may have adverse effects on renal function due to hemodilution and diminished oxygenation [25] but can be prevented through individualization and continuous therapy reassessment [26]. On the contrary, excessive oxygenation that leads to hyperoxia has been linked with further enhancement of ROS production and oxidative stress in patients with acute lung injury [27] and systemic inflammatory response (SIRS) [28].

The endothelial isoform of nitric oxide synthase (eNOS) is the main source of NO production from arginine and oxygen that is essential for the normal endothelial function and vascular tone, the prevention of platelet aggregation, and presenting anti-inflammatory properties [16, 25]. The “uncoupling” phenomenon is met when eNOS is deprived of its cofactors (i.e., calmodulin and tetrahydrobiopterin) and results in the oxidation of oxygen and the release of superoxide [29] that acts as a free radical adding on to oxidative stress. The above phenomenon takes place in inflammatory situations (such as sepsis) where there is an incremental cellular NO release (and oxygen consumption) and is mediated by the action of inducible nitric oxide synthase (iNOS). According to a theory, the heterogeneous iNOS expression in AKI that leads to focal increase of NO levels and is further enhanced by microcirculatory dysfunction results in the perpetuation of regional oxygen deprivation [30]. Thus, kidney damage not only is maintained but also expands. The iNOS-dependent inhibition of eNOS deteriorates endothelial function further shaping a triangle among ROS, NO, and oxygen [29, 30] in the pathophysiology of AKI and oxidative stress.

#### 1.1.2. Toll-Like Receptors (TLRs) and Damage-Associated Molecular Pattern (DAMPs)

TLRs are transmembrane, pattern recognition receptors, and currently, there have been about 10 recognized subtypes in humans [31]. DAMPs are endogenous molecules that may either initiate immune response or act as proinflammatory mediators (the latest are occasionally called alarmins) [32]. They are presented to the immune system after cellular lysis, scheduled exocytosis, or after the release of enzymes' matrix [33]. Apart from DAMPs, TLRs recognize pathogen-associated molecular pattern (PAMPs) (peptidoglycan and lipopolysaccharide from pathogens). Macrophages, endothelial cells, dendritic cells, and lymphocytes express TLRs. Kidney mesangial and tubular epithelial cells express TLR1, TLR2, TLR3, TLR4, and TLR6. Once a ligand is bind on the receptor, with the complicity of factors such as myeloid differentiation factor 88 (MyD88) and toll-receptor activator of interferon (TRIF), endogenous pathways are activated (nuclear factor kappa-B and mitogen-activated protein kinase pathway) and result in inflammation and interferon production [31, 34].

In 1994, Matzinger introduced the theory of “danger” that is sensed by the immune system, it does not necessarily originate from pathogens, and it has the ability to enhance or fire innate immune response so that the threat is sufficiently defeated [35]. DAMPs are the triggering factors for this process and come from endogenous, damaged cells, usually including proteins. In AKI, heat shock proteins (HSPs) and high-mobility group box-1 (HMGB-1) protein are the most common but several others have been suggested as well [33, 36]. According to accumulating evidence during oxidative stress, TLR activation from DAMPs further enhances the incremental release of the latest as it was shown with HSp70 and TLR2/TLR4 in an animal model during IRI [37]. On the contrary, the origin of HMGB-1 is not that clear. Evidence from in vitro studies in hypoxic hepatocytes is in favor of ROS regulation on HMGB-1 release with prerequisite functional TLRs [38]. As derived from the aforementioned evidence, there is an ambiguous relationship between DAMPs and TLR activation in oxidative stress. Also, the release of DAMPs is partly determined by TLRs who are the main regulators of overall immune answer in oxidative stress [33]. The magnitude of inflammation-oxidative stress complexity is yet to be revealed and translated.

#### 1.1.3. Autophagy in AKI

Autophagy is a continuous, catabolic process conserved through evolution that takes place at a cellular level [39]. It is generally described as a “housekeeping” process and aims at the removal of damaged and dysfunctional molecules as well as at the enhanced response to acute situations such as nutrient deficiency, ensuring the recycling of components for protein and energy synthesis and the elimination of toxic material [40]. Fundamental for the initiation of autophagy is the expression of the autophagy-related genes (ATG) that were first discovered in yeast, with the produced proteins being subjected to multiple posttranslational modifications that regulate the final outcome [41]. According to evidence from animal models and clinical trials, the ATG proteins increase in AKI. In particular, ATG proteins that augment in AKI with tubular dysfunction are microtubule-associated protein light chain 3 (LC3) and Beclin-1 [42, 43]. The first step is the formation of an intracellular, double-membrane organelle called phagophore that after the sequestration of the target turns into autophagosome and with the subsequent lysosomal fusion becomes the autolysosome that with the intermediary action of lysosomal enzymes will lead to the degradation of the contained cytoplasmic components in order to provide matrix for “recycling” [44]. The process is complete after lysosomal reformation and the inhibitory effect on autophagy of mammalian target of rapamycin receptor (mTOR) [39, 40]. Nevertheless, there are pending issues regarding the further clarification of the complicated signaling pathways in autophagy, their selectivity, and regulation [39].

In AKI, hypoxic damage in tubular epithelial cells is a potent stimulus for autophagy [45] that is generally considered beneficial and nephroprotective, preventing further structural compromise, especially at the S3 segment of the proximal tubule that is vulnerable to oxygen deprivation [46]. In AKI, apart from hypoxia, the increased ROS production due to inflammation and oxidative stress causes mitochondrial depolarization and dysfunction that through the PINK1/Parkin (PTEN-induced putative kinase protein 1) and the BNIP3/NIX/FUNDC1 pathway lead to selective mitochondrial autophagy (“mitophagy”) [24, 44]. Contradictory opinions exist and claim that autophagy can be deleterious promoting cellular apoptosis, adding on to the renal injury [47–49].

#### 1.1.4. Microvascular Dysfunction

Under normal circumstances, outer medulla is perfused with about half blood flow compared to cortex and the consequent partial oxygen pressure is 10–20 mmHg and 50 mmHg [50]. Thus, outer medulla is an especially vulnerable zone to circulatory disturbances and hypoxia. During AKI, the sustained renal perfusion through normal blood flow from renal artery does not secure the unhampered function of the complex renal microvasculature. Evidence data prove the existence of focal hypoxemic renal tissue in AKI [51] that add on to our comprehension regarding the pathophysiology of AKI [52]. In oxygen deprivation, anaerobic glycolysis is enhanced, lactic acid is accumulated, mitochondrial dysfunction is enhanced, and production of ROS and superoxide is upregulated [53]. The injury expands after reperfusion that is characterized by inflammatory response with leukocyte and complements activation that progresses to an oxidant environment that cannot be counterbalanced by antioxidant mechanisms [54] and uneventfully leads to excessive cell death [53].

Endothelium holds a crucial role regarding the expansion of inflammation, through expression of adhesion molecules such as selectins [55], the intracellular adhesion molecule-1 (ICAM-1) [56], and CX3CL1 (fractalkine) [57] that regulate inflammatory cell recruitment. The effect on vascular wall, along with the partly specified changes on glycocalyx [58], is increased permeability that in AKI, is expressed as proteinuria [58, 59].

### 1.2. Prediction of AKI by Oxidative Stress Biomarkers in Critically Ill Patients

A number of obstacles have hampered the investigation of the role of oxidant injury in multiple organ failure and AKI in critically ill patients. Among them is the fact that oxidative stress might be a focal, instant response resulting to the lack of stable, specific oxidative stress biomarkers that can be measured accurately and noninvasively in these patients [16]. Nevertheless, prevention of AKI requires among others the recognition of high-risk patients and early diagnosis based on accurate predictive tools. After the recognition of serum creatinine inadequacy in the prediction of AKI due to the variability of measured levels (based on age, gender, race, and muscle mass) with low sensitivity and specificity [60], novel plasma and urine biomarkers have been introduced. In the meantime, along with the enhanced comprehension of novel biomarker characteristics (for details refer to [61–65]), there is accumulating evidence concerning the predictive value and the clinical applicability of these molecules.

Oxidative stress can be assessed by indirect methods which can measure the stable by-products of ROS activity on biomolecules. In the setting of critical illness, the most commonly measured markers of oxidative stress are isoprostanes, hydroxynonenal and lipid peroxides, chlorinated compounds, oxidized glutathione, nitrated and oxidized proteins, and malondialdehyde detected as thiobarbituric acid reactants (TBARs) [15]. Among them, some biomarkers have been investigated in order to predict the occurrence of AKI in severely ill patients with different results. In an observational cohort study in ICU patients with severe sepsis, Ware et al. found that plasma levels of F2-isoprostanes and isofurans were associated with acute hepatic, coagulation, and renal failure [66]. Liver-type fatty acid-binding protein (L-FABP) has been considered as an important cellular antioxidant during oxidative stress by maintaining low levels of free fatty acids in the cytoplasm of tubular cells through facilitation of intracellular metabolism and excretion in urine. In a number of studies, urine L-FABP has been able to reliably predict the occurrence of AKI and death in ICU patients [64]. Recently, Costa et al. found that erythrocyte superoxide dismutase (SOD1) activity could play a role as an early marker of septic AKI and could be seen as a new research avenue in the field of biomarker in AKI [67].

According to robust evidence, an AKI-specific biomarker is the neutrophil gelatinase-associated lipocalin (NGAL) that can be measured in both plasma and urine [68, 69]. NGAL is a multifaceted protein that is rapidly induced and released from the injured distal nephron—among others. It has the ability to scavenge iron whose role is crucial for bacterial survival and is an important component to free radical generation. Thus, NGAL levels have been implicated in various types of organ injury, including myocardial infarction, cancer, sepsis, and AKI [70]. Apart from bacteriostatic effects [71], the protection against oxidative stress damage has been suggested [71, 72], while the exact antioxidative mechanisms of NGAL are still under question. There are data in favor of the upregulation of endogenous antioxidants such as SOD1 and SOD2 as well as HO1 levels [73, 74]. According to a 2009 meta-analysis in 8500 critically ill patients, the area under the curve (AUC) for the prediction of AKI (12 hours earlier) reached 0.85 for plasma NGAL and 0.86 for urine NGAL, superior to the predictive value of serum creatinine levels and eGFR, with sensitivity of 81–96% and specificity of 51–68% [75]. Urinary kidney injury molecule-1 (KIM-1) and interleukin-18 (IL-18) are suggested as good markers for the prediction of progressive AKI [75]. The high diagnostic value of IL-18 in AKI (odds ratio (OR): 5.11, AUC: 0.77) [76] is not corroborated by equal prognostic significance in critically ill patients [77], and the careful interpretation of urine IL-18 levels is highly recommended. KIM-1 was attributed with a good prognostic value of AKI development after cardiac surgery with high sensitivity 92–100% and AUC 0.78–0.91 [78], while the persistent elevation of urine KIM-1 levels might correlate with poor prognosis [79]. What should be mentioned is that NGAL, IL-18, and KIM-1 are inflammatory mediators that increase in inflammatory situations regardless of the presence of AKI and are indivisible parameters concerning their assessment in the prediction of AKI [65].

Recently, tissue inhibitor of metalloproteinase 2 (TIMP-2) and insulin-like growth factor-binding protein 7 (IGFBP-7) have been investigated as predictive urine biomarkers of AKI in high-risk patients [80, 81]. Both TIMP-2 and IGFBP-7 are cell cycle arrest biomarkers as they have been implicated in the G1 cell cycle arrest phase during the very early stages of cellular stress. It has been shown that renal tubular cells go through this G1 cell cycle arrest phase following stress due to a number of different causes. Specifically, the SAPPHIRE study assessed the urine product of TIMP-2 and IGFBP-7 and concluded that it is superior in the prediction of KDIGO stage 2-3 AKI compared with the rest of the biomarkers of the study, even NGAL and KIM-1 (*p* < 0.002) in critically ill patients [80]. Further analysis in the SAPPHIRE and OPAL study cohorts has set cut-off values for risk stratification of AKI with high-risk patients when TIMP-2 × IGFBP-7 is over 0.3 and the highest risk for patients with product value is over 2 [81]. Nevertheless, in persisting AKI that is equal with the ongoing damage, the levels of TIMP-2 × IGFBP-7 product remain elevated indicating the maintenance of cell cycle arrest (in G1 phase) that may uneventfully lead to failure of recovery and renal fibrosis [82]. Thus, the potential selective intervention in the activation and disruption of cell cycle might be beneficial for renal protection.

### 1.3. Clinical Evidence in AKI Prevention by Targeting Oxidative Stress

Albeit evidence for the role of oxidative stress in the pathogenesis of AKI originating mainly from experimental models and distinctive pathways remains obscure, the idea that controlling oxidative stress in patients with AKI may prevent or attenuate the severity of cellular injury has been explored in the clinical setting. Existing clinical evidence in this field, regarding critically ill and ICU patients, comes from small cohorts and studies. Nevertheless, the scavenging of free radicals in order to avoid the provocation of chain reactions that will lead to regional or generalized oxidative stress demonstrates great interest.

Anesthetics have been suggested as potential oxidative stress scavengers and in particular SOD mimetics (sodium pentothal and propofol) and lidocaine, when used in critical care practice [83]. N-acetylcysteine (NAC) as shown by in vitro studies acts as a direct scavenger of OH^−^ mainly, but when administered orally, the bioavailability is low and even untraceable. The antioxidant action of NAC is mediated by the induction of glutathione synthesis [84]. Data from trials in humans imply that NAC reduces the incidence of AKI after contrast media administration (*p* = 0.02) [85], but the direct intravenous administration of glutathione has been shown to be superior as regards renal protection against contrast-induced nephropathy compared to NAC per os [86].

Apart from the first-line treatment in lipid-lowering therapy, HMG-CoA reductase inhibitors, globally known as statins, participate further in vascular endothelium function preservation through upregulation of eNOS, thus increasing the available NO and contribute to the restriction of free radical generation from lipids' oxidation [87, 88]. In this direction, results from cohort studies concerning severe illness are in favor of the benefits of statins in the protection of renal function after percutaneous coronary angiography [89], acute coronary syndrome [90], and IRI [91]. On the contrary, a Cochrane database meta-analysis on the prevention of AKI with statin administration prior to major surgery failed to show reduction in AKI incidence for critically ill patients undergoing surgery with cardiac bypass [92].

Ischemic preconditioning (IPR) was introduced in 1986 by Murry et al. in an animal model that sustained brief ischemic episodes before a major ischemic event and resulted in a beneficial outcome for the organ [93]. In 1993, Przyklenk et al. described a slightly different model of ischemic preconditioning (remote and rIPR) [94] that has been further modified and is currently followed, when the direct approach to the involved organ is not feasible. The underlying mechanisms are notably complex and not totally unraveled. In brief, after the main stimuli (ischemia) is withdrawn, a series of responses take place (neural, humoral pathway, and systemic anti-inflammatory response) with the final receiver being the mitochondrion [95]. The subsequent opening of the ATP-dependent mitochondrial potassium channel prevents the opening of the mitochondrial permeability transition pores (MPTP) that enhances the stability of its membrane [96] and the survival after IRI [97].

Generally speaking, rIPR concerns clinical practice and especially critical care when it comes to scheduled procedures that carry a significant burden for homeostasis and are closely related with the induction of systematic inflammation and oxidative stress, such as cardiac surgery procedures. The highly vulnerable to hemodynamic imbalance renal cortex and its complex microvasculature are affected by rIPR. According to a recent review (2016) by Ho et al., who included 17 clinical trials that examined the renal outcome in different rIPR cases, a notable renal protection is shown in 12/17 of the trials with no significant deviations in the rest of the trials [98].

### 1.4. Therapeutic Interventions and Future Perspectives

In the current clinical practice, there is a lack of standardized preventive measures against AKI in severely ill patients apart from general suggestions for maintenance of fluid and electrolyte balance, avoidance of unnecessary exposure to potentially nephrotoxic agents, and continuous clinical monitoring [99, 100]. Earlier efforts to show benefit in renal outcome in critical care setting through administration of low-dose dopamine in continuous infusion have shown a temporary benefit in urine output [101], but with no significant protection against the development of AKI, the prevention of RRT, and mortality according to meta-analyses [102–104]. In the same patient group, fenoldopam seems to be superior compared to dopamine in the improvement of serum creatinine levels when renal dysfunction is present [105] and according to a meta-analysis in 1290 patients, fenoldopam administration reduced the need for RRT support and intensive care unit hospitalization [106].

As regards interventional measures in order to control oxidative stress response in critically ill patients, the interest has been focused on macro- and micronutrients and the correlation of their levels with patients' general clinical course and outcome and not just with the prevention or therapy of AKI. The early recommencement of enteral versus parenteral feeding in ICU patients (even before 48 hours of hospitalization) that contributes on the maintenance of normal intestinal microflora has been correlated with better survival and less infections [107]. Nevertheless, the optimal dose that permits autophagy and provides the highest benefit for ICU patients has not been quantified yet [107, 108]. The supplementation of trace elements in critically ill patients has employed investigators and in particular the administration of thiamine, vitamin C and E, and selenium separately has been found to improve survival and reduce infectious complications [109]. On the contrary, no clear benefit on critically ill patients' survival was shown in a meta-analysis of 4 trials with zinc administration, neither a benefit in the duration of ICU stay [110]. In a meta-analysis of 21 randomized control trials, it was shown that the supplementation of trace elements correlated with reduction in the number of days with need for mechanical ventilation, but the establishment of clear conclusions regarding the best possible route of administration (enteral versus intravenous) was not feasible due to significant heterogeneity of the available data [111]. Even if this replenishment concerns relatively short periods, toxicity [109, 110] is to be kept in mind and appropriate measures should be applied in order to avoid it. In general, the substitution of more than 66% of the recommended daily allowance of vitamins A, C, and E has been shown to improve antioxidant capacity [112]. REDOXS (Reducing Deaths due to Oxidative Stress) study is a blinded randomized trial in 1223 critically ill patients that failed in meeting its original rationale and concluded that the administration of antioxidants and glutamine increased mortality [113]. Among the possible reasons are the doses chosen of the implemented therapeutic strategy and the potential toxicity that may have defined the final outcome [114]. Enteral administration of melatonin [115] and parenteral administrations of NAC plus deferoxamine [116] have been correlated with better total antioxidant capacity (TAC) in serum. As derived from the presented data, we are not yet capable to reach safe conclusions with clinical applicability as regards the initiation, dose, route, and duration of therapy for the aforementioned strategies.

As regards future perspectives, antioxidants targeted to mitochondria have been developed and the main axis of their action is through the electrical potential and the pH gradient of the mitochondrion that leads to the selective accumulation of these cationic molecules. Mito-vitE, MitoQ, MitoPBN, and MitoPeroxidase have the ability to prevent ROS generation and enhance mitochondrial survival [117–119]. The optimization of understanding the mechanisms of action has gained a lot of interest, as well as the enhancement of their chemical synthesis [120]. Unfortunately, current literature lacks in vitro or in vivo studies investigating the administration of antioxidant targeted molecules.

## 2. Conclusions

Acute kidney injury is a multifactorial clinical entity representing a major health problem. In critical care, AKI remains highly prevalent, complicating the clinical course of patients, extending the need for ICU hospitalization, requiring RRT, and carrying high mortality. Pathogenesis of AKI is complex and remains incompletely elucidated. Oxidative stress is involved in the pathogenesis of AKI and is characterized by complex, codependent mechanisms that progress to organ response and damage. More extensively, main experimentally explored mechanisms of oxidative stress involved in AKI summarize to ROS generation, NO depletion, DAMP generation and TLR activation, autophagy, and microvascular dysfunction. These mechanisms prevail over endogenous antioxidants and regulatory mechanisms so that physiological homeostasis is abolished and AKI is finally installed.

Prevention of AKI is essential and requires among others the recognition of high-risk patients and early diagnosis based on accurate predictive tools. In the setting of critical illness, the most commonly measured markers of oxidative stress are isoprostanes, hydroxynonenal and lipid peroxides, chlorinated compounds, oxidized glutathione, nitrated and oxidized proteins, and TBARs. Novel AKI-specific biomarkers available are NGAL, KIM-1, and levels of TIMP-2 × IGFBP-7 with accumulating evidence being in favor of their diagnostic and prognostic value. Further progress that will encompass in daily practice techniques allowing more accurate assessment of oxidative stress will further improve the prevention of AKI in critical care. Therapeutic interventions trying to control oxidative stress response in critically ill patients have been focused on macro- and micronutrients. Currently, there are encouraging results from the inhibition of oxidative stress via exogenous administration of antioxidants and methods as ischemic preconditioning, but no standardized therapeutic protocols exist. The role of antioxidant therapy requires further elucidation and attention in the care of critically ill patients and in AKI. The meticulous study and interpretation of available observational data and expansion of existing knowledge through well-designed interventional studies in the setting of critical illness are necessary.

## Figures and Tables

**Table 1:** Common causes and susceptibilities for AKI.

Sepsis	
Circulatory compromise (shock)	
Burns/trauma	
Cardiac surgery (especially with cardiopulmonary bypass)	
Major (noncardiac) surgery	
Nephrotoxic drugs	
Radiocontrast agents	
Poisonous plants/animals	
Volume depletion	
Advanced age	
Female gender	
Black race	
Chronic kidney disease	
Diabetes mellitus	
Cancer	
Anemia	

**Table 2:** Acute kidney injury stratification criteria.

AKIN		KDIGO		
Serum creatinine	Stage	Stage	Serum creatinine	Urine output
≥0.3 mg/dL increase or increase ×1.5–2 from baseline	**1**	**1**	×1.5–1.9 from baseline or ≥0.3 mg/dL increase	<0.5 mL/min/kg ×6–12 h
Increase ×2-3 from baseline	**2**	**2**	×2–2.9 from baseline	<0.5 mL/min/kg for ≥12 h
Increase > ×3 from baseline or sCreatinine ≥4 mg/dL with acute increase of at least 0.5 mg/dL	**3**	**3**	×3 from baseline or sCreatinine ≥4 mg/dL or renal replacement therapy or eGFR <35 mL/min/1.73m^2^ in patients <18 yo	<0.3 mL/min/kg for ≥24 h or anuria for ≥12 h

AKIN: Acute Kidney Injury Network; KDIGO: Kidney Disease: Improving Global Outcomes; GFR: glomerular filtration rate; ESKD: end-stage kidney disease.
